# Optical coherence tomography microscopy in experimental traumatic brain injury

**DOI:** 10.1002/jemt.23599

**Published:** 2020-10-03

**Authors:** Eugen Osiac, Smaranda Ioana Mitran, Cătălin Nicolae Manea, Alexandru Cojocaru, Gabriela‐Camelia Rosu, Mariana Osiac, Daniel Nicolae Pirici, Adrian Tudor Bălșeanu, Bogdan Cătălin

**Affiliations:** ^1^ Experimental Research Center for Normal and Pathological Aging University of Medicine and Pharmacy of Craiova Craiova Romania; ^2^ Department of Biophysics University of Medicine and Pharmacy of Craiova Craiova Romania; ^3^ Department of Physiology University of Medicine and Pharmacy of Craiova Craiova Romania; ^4^ Department of Informatics, Communication and Statistics University of Medicine and Pharmacy of Craiova Craiova Romania; ^5^ Department of Research Methodology University of Medicine and Pharmacy of Craiova Craiova Romania; ^6^ Department of Physics, Faculty of Science University of Craiova Craiova Romania

**Keywords:** animal model, optical coherence tomography, traumatic brain injury

## Abstract

Worldwide elderly traumatic brain injury (TBI) patients tend to become an increasing burden to the society. Thus, a faster and less expensive way of evaluating TBI victims is needed. In the present study we investigated if optical coherence tomography (OCT) could be used as such a method. By using an animal model, we established if OCT can detect cortical changes in the acute phase of a penetrating TBI, in young (5–7 months) and old (20–22 months) rats. Due to the long‐term evolution of TBI's, we wanted to investigate to what extent OCT could detect changes within the cortex in the chronic phase. Adult (7–12 months) male rats were used. Surprisingly, OCT imaging of the normal hemisphere was able to discriminate age‐related differences in the mean gray values (MGV) of recorded pixels (*p* = .032). Furthermore, in the acute phase of TBI, OCT images recorded at 24 hr after the injury showed differences between the apparent damaged area of young and aged animals. Changes of MGV and skewness were only recorded 48 hr after injury. Monitoring the chronical evolution of the TBI with OCT revealed changes over time exceeding the normal range recorded for MGV, skewness and kurtosis, 14 and 21 days after TBI. Although in the present study we still used an extremely invasive approach, as technology improves, less invasive and non‐harmful ways of recording OCT may allow for an objective way to detect changes within the brain structure after brain injuries.

## Graphical abstract

- Optical coherence tomography microscopy was used in an experimental model of traumatic brain injury to establish if the method could pick up differences in the acute and chronic phases of injury, in rats of different ages.
- A series of direct (mean gray value) and synthetic (skewness and kurtosis) parameters were used to compare the data sets.

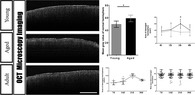

## 1 INTRODUCTION

Classic medicine sees traumatic brain injury (TBI) as a young adult pathology (Fujimoto, Pitris, Boppart, & Brezinski, 2000; Morganti‐Kossmann, Rancan, Stahel, & Kossmann, 2002; Sharma, Changoor, Monteiro, Colella, & Green, 2020); recent epidemiological data suggests that brain injury has a trimodal distribution, affecting children between 0 and 4 years old, young adults (15 to 19 years of age) and older adults (over 65 years) (Escobedo et al., 2013; Kisser, Waldstein, Evans, & Zonderman, 2017; Taylor, Bell, Breiding, & Xu, 2017). The last segment represents a true modern problem: as aging continues, elder people tend to accumulate co‐morbidities (Sinha et al., 2008). Especially in this group even domestic accidents have a tendency to generate long term complications (Mak et al., 2012) and have worse predictions compared with younger persons (Hukkelhoven et al., 2003; Livingston et al., 2005; Stitzel et al., 2008).

The 2016 World Health Organization report placed injuries among the first 10 global causes of death, killing 1.4 million people (Roth, Abate, Abate, & Collaborators, 2017). In Europe injuries are also the leading cause of death between the age of 15 and 44 years (Krug, Organization,, & Team, 1999). From all the different types of injury between 10 (Hyder, Wunderlich, Puvanachandra, Gururaj, & Kobusingye, 2007) to 69 million (Dewan et al., 2018) people are globally affected each year by TBI.

Despite increasing incidents and life threats, research into traumatic brain injuries is underfinanced, being, in 2015, the sixth field financed by the National Institutes of Health in 2015 (Coute, Panchal, Mader, & Neumar, 2017), but according to the Centers for Disease Control and Preventionh, having a similar death count as kidney disease (Taylor et al., 2017). Treatment strategies did not alter too much; doctors are still focusing on the prevention of secondary injuries: while primary brain injury—a direct result of the damage caused to the brain parenchyma by a violent trauma—is nonreversible, secondary injury can be partially prevented. The last one occurs in hours, even days after the primary injury and it can be limited by correcting hypoxemia, hypotension, anemia, hypoglycemia, fever or by surgery (Greer, Funk, Reaven, Ouzounelli, & Uman, 2008; Reaven, Lovett, & Funk, 2009; Rosonke & Legome, 2006). Some studies showed that, alone or combined, pre‐hospital lesions, hypoxemia, hypotension, or pyrexia are predictors for mortality and/or disability (Jones et al., 1994); others concluded that even a single episodic drop in systemic blood pressure under 90 mmHg is an independent risk factor for both death and disability (He et al., 2016; Ker, Perel, Blackhall, & Roberts, 2008).

Often neglected in classical medicine, elderly TBI patients are going to be an increasing burden to the society considering the worldwide aging population. Although aged patients represent only 10% of all TBI patients, they account for 50% of all TBI related deaths (CDC, 2006); moreover, there are studies showing a significantly increased mortality to patients who sustained multiple traumatic injuries over the age of 56 years (Kuhne, Ruchholtz, Kaiser, & Nast‐Kolb, 2005). Other studies showed different age ranges but all agreed that, no matter the severity of the injury, mortality is higher among elder than among younger individuals (McIntyre, Mehta, Aubut, Dijkers, & Teasell, 2013; Røe, Skandsen, Manskow, Ader, & Anke, 2015). Furthermore, older survivors of TBI have a lower long‐term survival rate than their age‐matched noninjured peers, indicating that TBI predisposes old people to premature death (Flanagan, Hibbard, Riordan, & Gordon, 2006); unfortunately the TBI also severely increases the predisposition of elder people to depression (Menzel, 2008).

Even though numerous research articles on TBI were written, most of them were carried out on young adult and adolescent rats or mice. Nevertheless, a few studies were realized on old animals too and showed that cellular response of microglia and astrocytes had a more intense and prolonged response to TBI in aged animals compared with younger ones (Sandhir, Onyszchuk, & Berman, 2008). This tendency could correspond to a more intensive gliosis and partially explains the poor outcome of aged patients compared with younger ones. More interesting is the fact that aged animals showed an accentuated neurodegeneration, prolonged edema during the acute phase of the injury, increased blood–brain barrier disruption and, as a direct consequence, higher functional loss (Onyszchuk, He, Berman, & Brooks, 2008). It seems that, because the aged brain has the capability of mounting a cytoproliferative response to injuries, there is a dysregulation in the timing of the cellular and genetic response for the animals, compromising their functional recovery (Petcu et al., 2008).

Transport and mobilization of the patient involve huge costs and risks. Monitoring the evolution of a TBI with efficient techniques, such as magnetic resonance imaging (MRI) and computer tomography (CT), is also not always possible. Therefore, newer, cheaper high resolution and faster techniques such as optical coherence tomography (OCT) could be an appropriate imaging method to evaluate TBI in the future. Moreover, having an almost cellular resolution it may be useful in evaluating, monitoring and elucidating morphological and physiological processes occurring after injuries.

Although OCT was introduced to medical research more than two decades ago for identifying structural and functional investigations of the eye in ophthalmology (Fenolland, Puech, Baudouin, & Labbe, 2013; Fercher, Hitzenberger, Drexler, Kamp, & Sattmann, 1993; Swanson et al., 1993), it has been applied and proved to be an efficient tool in cardiology (Tearney et al., 1996) and dermatology (Welzel, 2001). New applications of OCT in gastroenterology (Chen et al., 2008; Gerard & Jr, 2017; Osiac, Saftoiu, Gheonea, Mandrila, & Angelescu, 2011; Zhang, Chen, & Isenberg, 2009) or neurology (Boppart, Brezinski, Pitris, & Fujimoto, 1998; Radhakrishnan & Srinivasan, 2013; Srinivasan et al., 2013; Srinivasan, Radhakrishnan, Jiang, Barry, & Cable, 2012; Zhang et al., 2011) captured researchers' attention in the last years, and lately neuroscientists have tried to use it (Beckmann et al., 2019; Dolezyczek et al., 2020; Osiac et al., 2014; Osiac et al., 2014; Sfredel et al., 2018). In the current work we are trying to establish if OCT measurements can be considered in the evaluation of TBIs.

## 2 MATERIALS AND METHODS

### 2.1 Animals

For this study young (*N* = 20, 5–7 months, with an average body weight of 410 g), adult (*N* = 20, 7–12 months, with an average body weight of 490 g) and old (*N* = 20, 20–22 months, with an average body weight of 675 g) Sprague Dawley (SD‐CD) male rats were used. The animals were housed under standard laboratory conditions: 12 hr light‐12 hr dark cycle, 21°C temperature, relative humidity of ~55%, free access to food and water. All procedures were carried out in accordance with European Union Directive 86/609/EEC regarding animal care and experimentation. The experiments were performed according to the ethical guidelines of the national animal protection law and were authorized by the ethical committee of the University of Medicine and Pharmacy of Craiova (no. 12/2020).

### 2.2 Anesthesia and surgery

For anesthesia an intra‐peritoneal cocktail of Ketamine (100 mg/ml) and Xylazine (20 mg/ml) was used. The rats were weighed in order to avoid overdosing with Ketamine before any invasive maneuvers. The depth of anesthesia was tested every 10–15 min by toe pinching reflex evaluation.

Anesthetized rats were placed on a heating pad for the duration of the procedure. Using a betadine solution, the skin covering the skull was disinfected. A longitudinal insertion was made in such way that a good visualization of the parietal bones, bregma, lambda, and lambda suture was achieved. All additional subcutaneous fat was removed. After the skull was cleaned, a 2 by 1 mm longitudinal craniotomy on the right parietal bone was made, starting 1 mm from the sagittal suture and stopping 1 mm before the lambdoid one. The craniotomy was always within the parietal bone. During drilling, every 15–20 s NaCl 0.9% or PBS were added on the drilled surface, ensuring the cooling of the bone. The craniotomy was performed in such way that the internal lamina of the parietal bone remained intact, in a similar manner to thinning bone procedure (Marker, Tremblay, Lu, Majewska, & Gelbard, 2010). After the partial craniotomy was completed, a TBI was done by inserting a blunt instrument (2 mm length and 0,5 mm width) about 5 mm into the brain. This ensured both the dura's disruption and the cortical compression by the previously intact internal lamina, thus better mirroring a penetrating TBI (Cernak, Wing, Davidsson, & Plantman, 2014; McColl et al., 2018). After retrieving the instrument, the skin was sutured and betadine solution was applied.

For the sham group, the same procedure was applied, except the blunt object insertion. After craniotomy and trauma application, the rat did not undergo other surgical procedures. Four hours before the surgery, all animals received Carprofen (5 mg/kg), to minimize postsurgery pain.

### 2.3 Study design

**FIGURE 1:**
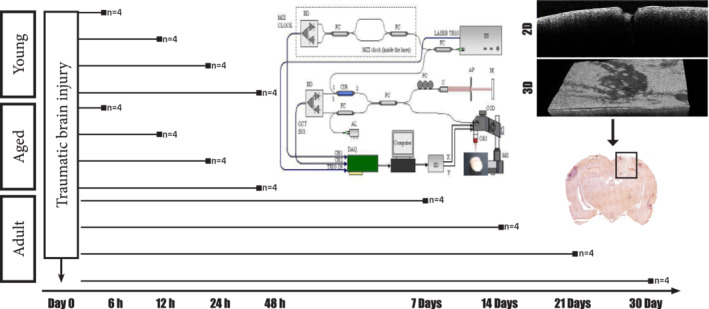
Basic experimental setup. Young, old and adult rats were subjected to a penetrating traumatic brain injury and OCT imaging was performed at different time points. Additionally, immunohistochemistry was performed to try to establish a correlation between the cellular response and OCT findings [Color figure can be viewed at wileyonlinelibrary.com]

This study was focused on establishing if OCT can be used to evaluate and monitor cortical changes produced by TBI. Due to the multimodal evolution of TBI's the study needed to address separately changes that might be detected in the acute phase (first 48 hr after TBI) and chronic one (7 to 30 days after TBI) (Figure 1).

The first part of the study was focused on the capacity of OCT to distinguish subtle morphological changes that occur in the young and elderly brain, during the acute phase of a TBI (Onyszchuk et al., 2008; Rowe et al., 2016; Sandhir et al., 2008). As such OCT images of young animals with brain lesion were compared with OCT images obtained from old animals with similar lesions. This was aimed to evaluate the OCT power of detecting changes that are known to be appearing in older nervous structures, compared with the young ones (Rowe et al., 2016). The measurements were done at an interval of 6 hr for the first 2 days after TBI.

The second part of the study was focused on evaluating if OCT imaging can detect the diffused morphological changes that accompanies TBI in the chronic phase. For this part of the study, we used only adult animals, as young animals have a longer longitudinal evolution of cortical damage (Zhao, Wang, Gao, & Chen, 2018). OCT brain changes that usually occur late after a traumatic event were therefore targeted. OCT imaging sessions were made 7, 14, 21, and 30 days after TBI.

### 2.4 OCT imaging

OCT is an optical imaging method that is using a partial coherent source in order to set the spatial resolution and based on an interferometric analyses of the reflected light on the investigated tissue it provides safe, high resolution and fast imaging (Fercher et al., 1993; Fujimoto et al., 2000; Osiac et al., 2011; Swanson et al., 1993). While interference is not a linear process, the obtained signal in such a set‐up is depending (but not linear) on the amount and the distribution of the scattered photon (also a statistic process) inside the analyzed sample which is also depending on the structure and characteristics of the sample. For this reason, statistical parameters as MGV, skewness or kurtosis of the received signal, parameters which are connected with the amount (for MGV) and distribution (for skewness and kurtosis) of the involved photons will provide a fair insight of the results of the method. Due to technological limitations, OCT imaging is, for the time being, highly difficult through the skull of the animal. As such, for the sake of simplicity, OCT imaging was done after the animals were transcardially perfused with room temperature phosphate buffered saline followed by paraformaldehyde 4%. The whole brain has been removed from the skull and dried for 10 min at room temperature, to avoid unnecessary reflections due to liquids present at the surface of the cortex. Excess water was removed by gentile tissue compression of the fixed brain. We used an OCT system (OCT1300SS, Thorlabs) powered by a swept laser source with central wavelength of 1,325 nm, a spectral bandwidth of 100 nm and an average power of 12 mW. The device makes possible 2D and 3D scan (with an axial‐scan rate of 16 kHz). Axial and lateral resolutions of this system are 12 μm and 15 μm (in air), respectively. The power on sample was ~5 mW. The signal detection of the OCT device is due to a CCD camera (charge‐coupled device camera). A special design controller, which allows rotation at different angle and holds the sample, was used in our experiments. Healthy tissue was also included in lesion scanning. Coronary pictures (5 × 2 mm) were sampled over a length of 3 mm, generating at the end a stack of 1024 × 512 × 512 pixels (width x depth x length).

### 2.5 Immunohistochemistry

After intra‐cardiac perfusion using NaCl 0.9% to wash the blood and paraformaldehyde 4% to fix the tissues, the removed brains were further fixed in 10% neutral buffered formalin (NBF) for 2 days, at room temperature, then processed for paraffin embedding and sectioning on a HM355S rotary microtome (Thermo Scientific Inc., Walldorf, Germany). Sections, 4 μm‐thick, were further processed for classical hematoxylin–eosin staining, as well as for immunohistochemistry (IHC). For IHC, the sections were rehydrated, subjected to antigen retrieval by microwaving in citrate buffer pH 6 for 20 min, incubated in a 1% hydrogen peroxide solution for 30 min in order to quench the endogenous peroxidase, and kept for another 30 min in 3% skimmed milk in PBS for blocking unspecific antigenic binding sites. The sections were then incubated at 4°C for 18 hr with the primary antibody (goat anti‐ionized calcium‐binding adaptor molecule 1 [Iba1], Abcam [Cambridge, UK], 1:1.000; rabbit anti‐Glial Fibrillary Acidic Protein [GFAP], Dako [Glostrup, Denmark], 1:30.000), and the next day the signal was amplified for 30 min with specific anti‐species peroxidase polymer detection system adsorbed for rat immunoglobulins (Nichirei Bioscience, Tokyo, Japan). The signal was finally detected with 3,3′‐Diaminobenzidine (DAB) (Dako) and the slides were coverslipped in DPX (Sigma‐Aldrich, St. Louis, MO). Negative controls were obtained by omitting the primary antibodies.

All the slides were scanned with a ×20 plan apochromat objective on a Nikon 90i microscope (Nikon Instruments Europe BV, Amsterdam, The Netherlands) equipped with a Prior OptiScan ES111 motorized stage (Prior Scientific, Cambridge, UK), a Nikon DS‐Ri2 CMOS 16 Mp color camera and the Nikon NIS‐Elements Advanced Research mapping and control software. Images were saved as tiff files and Nikon n2d proprietary format files.

### 2.6 Data analysis

All acquired images were analyzed using ImageJ. Sets of 15 consecutive images were converted into stacks. No adjustment was previously done. After manually selecting the core of the lesion for each image area, we calculated the mean gray value (MGV), the skewness (measure of the asymmetry of the probability distribution of a real‐valued random variable about its mean) and the kurtosis (it is any measure of the “peakedness” of the probability distribution of a real‐valued random variable [Westfall, 2014]) for each of the selected regions of interest. Data generated in this manner were used in our statistical analysis. Repeated ANOVA measurements were used to analyze group results, while independent data sets were analyzed with Mann–Whitney *U*‐test. Statistical significance was accepted for *p* < .05, ensuring a level of confidence of 95%, with statistical significance displayed as follows *: *p* < .05, **: *p* < .01 and ***: *p* < .001. Results were expressed as mean ± *SD*, unless indicated otherwise. For the chronic phase of the TBI all parameters were plotted against the values recorded from the contralateral hemisphere.

## 3 RESULTS

All rats used in this study, both acute and chronic experiments, survived the TBI procedure and the experimental period. None of the used rats displayed abnormal behavior or had clinical signs of infection following the surgery. In order to analyze the OCT results we determined area, mean gray value, skewness, and kurtosis of damaged cortex, as mentioned above. The region of interest (ROI) was manually selected for each image in the stack, to ensure the maximum sensibility of the OCT results.

### 3.1 OCT imaging can detect age‐related differences

**FIGURE 2:**
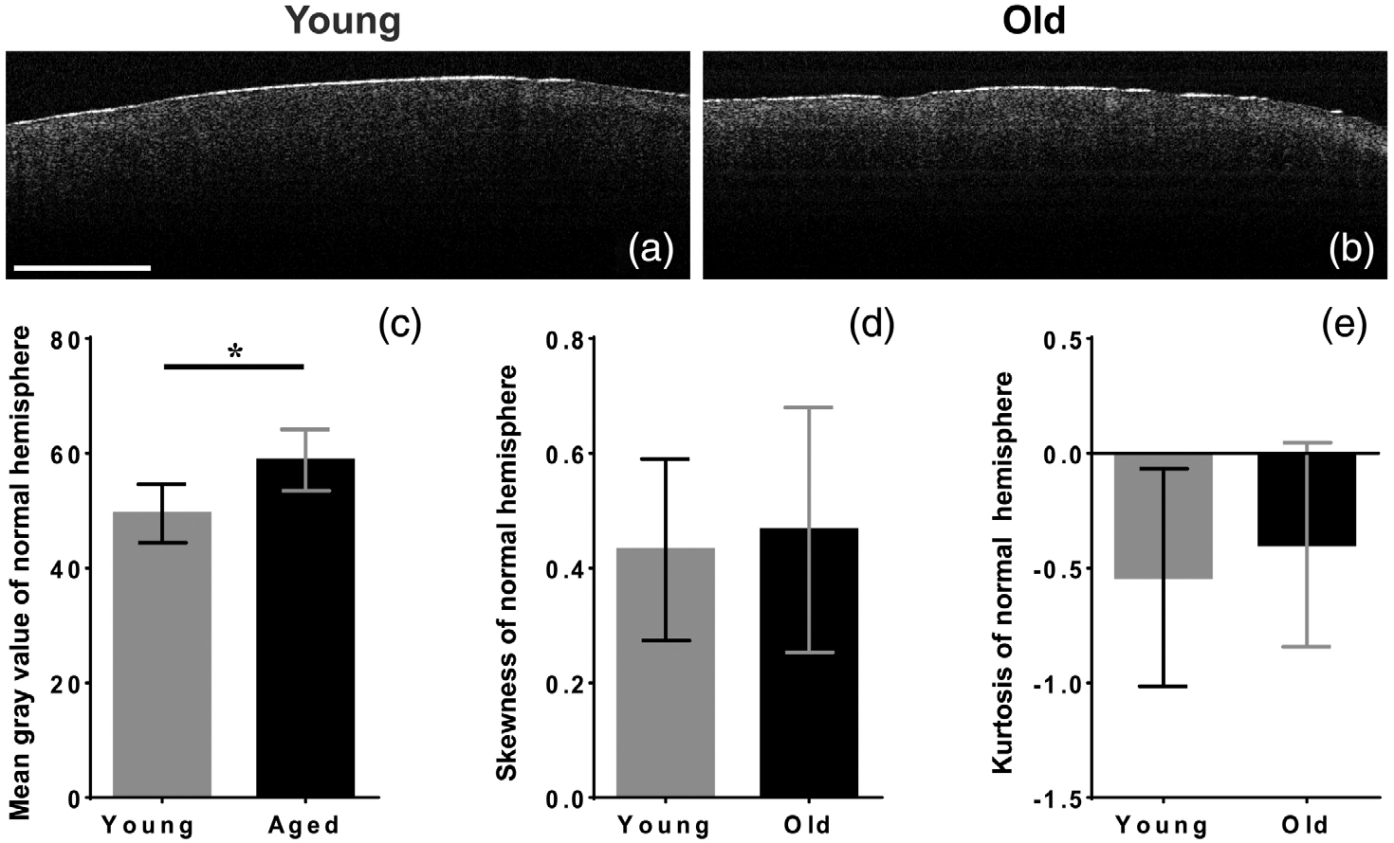
OCT scan of the contralateral hemisphere. Although, raw images (a and b) do not really seem to be different between young and old animals, the MGV found by measuring the intensity of recorded pixels, shows an increase in intensity in old animals compared with young ones, with no difference in symmetry between the groups (d and e)

Before starting measurements and establishing the potential for OCT investigation in TBI, we tested if image scanning of the cortex obtained from young and old animals generated different data sets. By scanning the normal ipsilateral cortex, we were able to establish normal values for MGV, skewness, and kurtosis. To our surprise, OCT images, obtained under the experimental conditions, were able to detect differences in MGV between young and old cortex (Figure 2c), with young animals having a lower MGV compared with aged ones (*p* = .032). When analyzing data distribution, results showed that both groups had a relatively normal distribution with no notable differences (Figure 2d,e).

### 3.2 OCT imaging can detect changes between young and old rats in the acute phase of TBI

**FIGURE 3:**
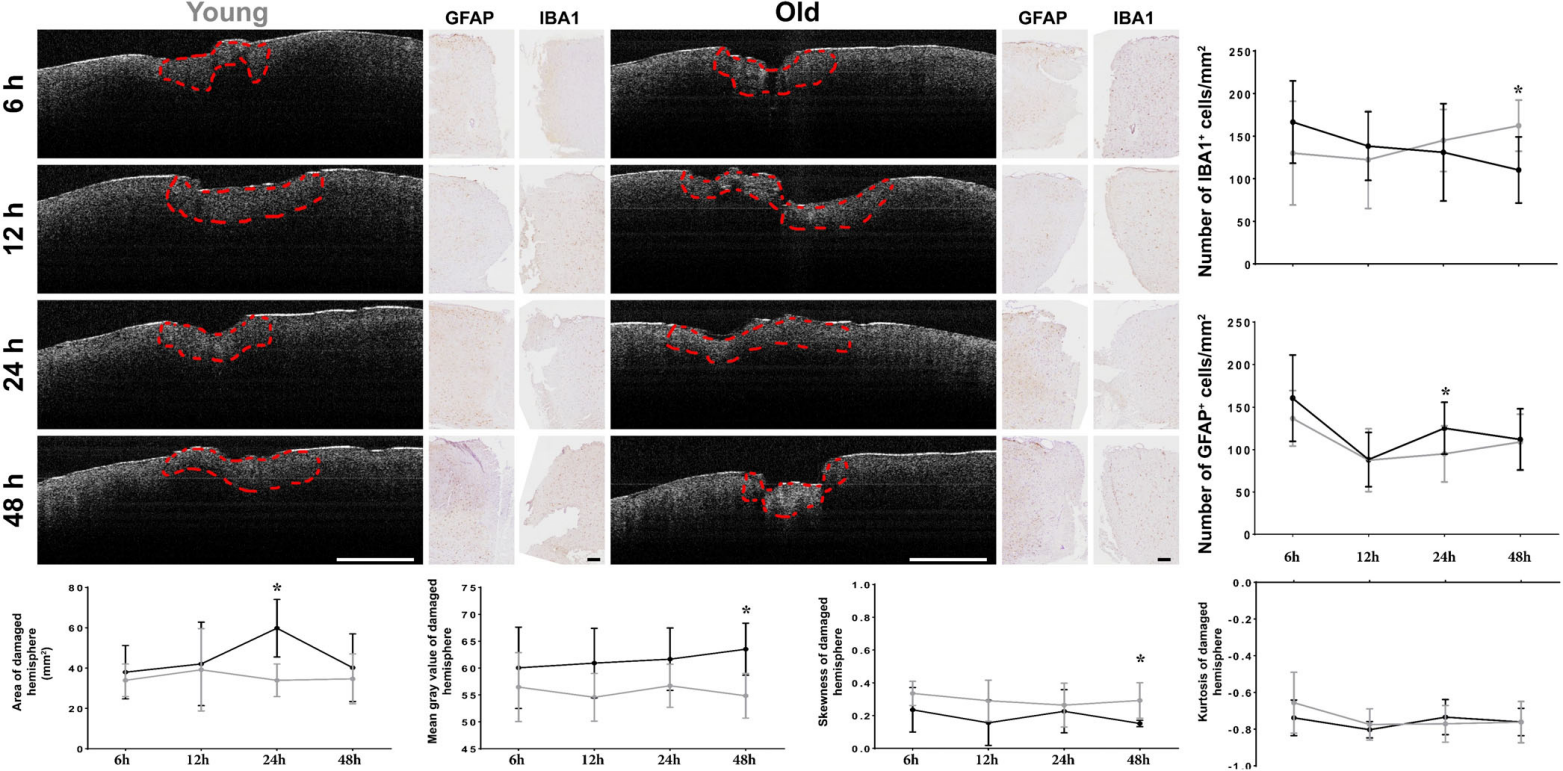
OCT scan and histological findings in the acute phase of TBI. Examples of OCT areas analysis are highlighted with red dashed line for both young and old animals, while the histological optical picture is presented for astrocytes (GFAP) and microglia/macrophages (IBA1) at the same time points. We were able to detect different cellular responses between young and aged animals when evaluating the cellular density of IBA1^+^ cells 48 hr after TBI, while GFAP^+^ cells seem to be more abundant in older animals 24 hr after TBI. In young animals the apparent damaged area seems to decrease 12 hr after the TBI while in aged animals this phenomenon can be seen 24 hr after the initial injury. The mean gray value of the images obtained using OCT was different between the two groups only 48 hr after the TBI, with a significant asymmetry at the same time point in regard to skewness. No difference can be observed in the kurtosis of the analyzed images. Scale bar 1 mm for the OCT images and 100 μm for the histological images [Color figure can be viewed at wileyonlinelibrary.com]

By using simple immunohistochemistry only two cellular differences were observed in the acute period of the TBI. An increase of GFAP^+^ cells in old animals 24 hr after TBI around the lesion site and an IBA1^+^ cells increase 2 days after in young ones (Figure 3a). The OCT measurements were also able to identify some parameters showing differences in the young and old response to TBI.

The apparent damaged area measured using OCT, between young and aged, was not different for the duration of the acute phase (Figure 3), except 24 hr after the TBI. At the time of the first measurement, 6 hr after TBI, the lesion area was slightly larger in older animals (37.971 ± 13.222 mm^2^) compared with the young ones (33.931 ± 8.091 mm^2^), slowly increasing within the first 12 hr for both young and old animal groups. After 12 hr, the apparent lesion observed with OCT measurements seemed to decrease in young animals, while in older animals this process seemed to be delayed, as the decrease was evident 48 hr after TBI. The only major difference was measured 24 hr after TBI when aged animals seemed to have larger damaged areas compared that young ones (*p* = .0113). By measuring MGV, a raw overlay can be obtained from the two groups. No difference was measured 6 hr after the TBI between old (60.04 ± 7.5) and young (56.46 ± 6.4) animals (*p* > .05). The MGV of old animals presented a slow increase that was different form the young group, 48 hr after TBI (*p* = .0165). No difference in MGV was detected at 12 and 24 hr after TBI. While MGV constantly increased in the older animals, for the young ones it fluctuated more, which could suggest a more dynamic response to injury in younger brain.

Measuring the asymmetry of the gray values obtained by OCT we found that skewness in the acute phase was different between the young and old animals only 48 hr after TBI (*p* = .0384). Although both groups tend to have high symmetry, older animals had almost perfect right–left distribution with the lowest skewness index being recorded 48 hr after the TBI (skew = 0.152). Regarding kurtosis, we observed no differences, with the two groups having almost identical “peakedness” of the probability distribution of a real‐valued random variable determined by OCT, with a marked more “flatten” pick compared that normal (Figures 3 and S1).

### 3.3 OCT can detect marked fluctuations in the chronic phase of TBI

**FIGURE 4:**
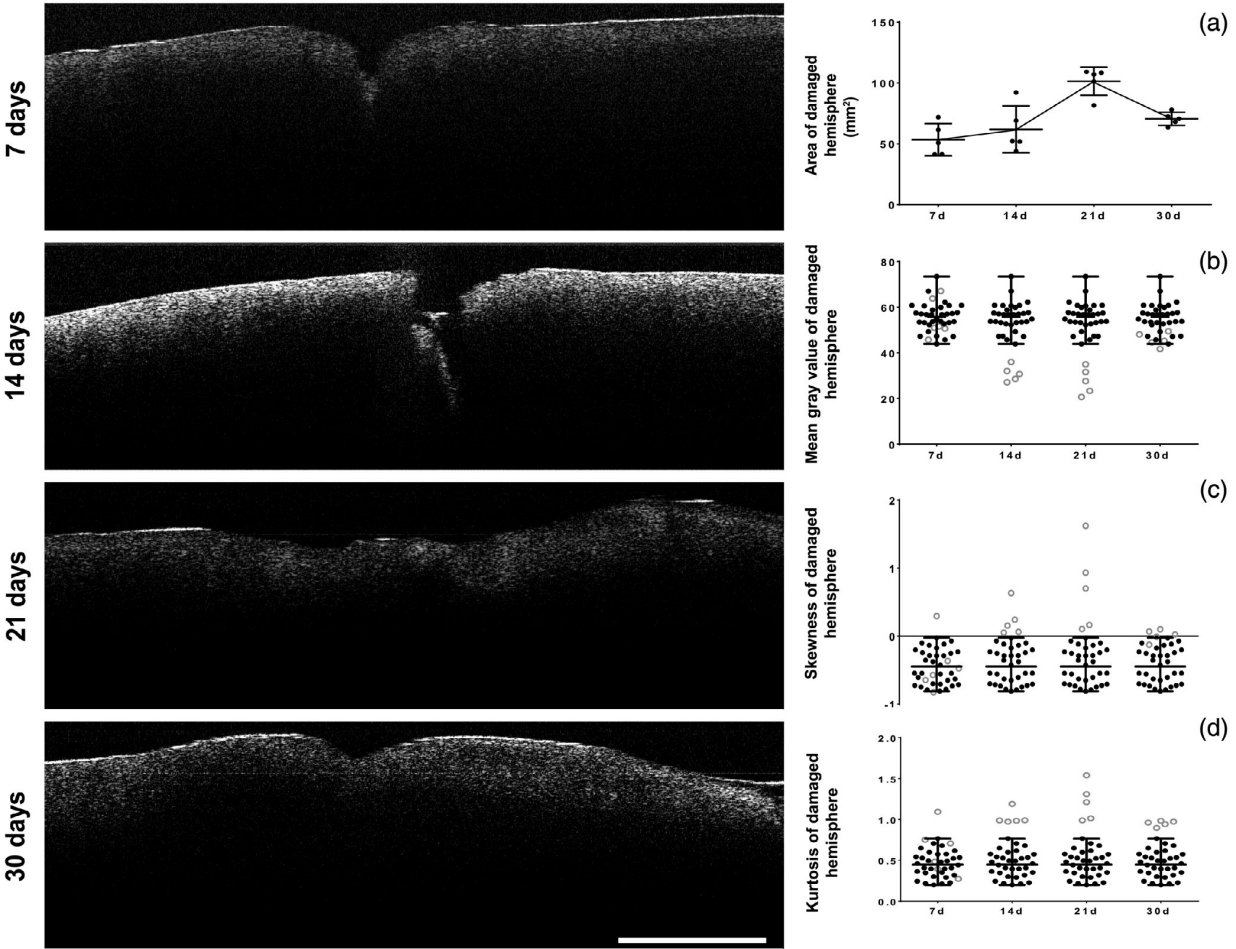
OCT imaging in the chronic phase of TBI. Optical scans can detect changes in the cortex of adult animals long after the initial injury. Changes in apparent damaged area could be observed using OCT over the chronic phase of TBI (a). By plotting the values and range of normal values obtained from the contralateral hemisphere against the values obtained in the ipsilateral one, we were able to establish that OCT imaging could be used in determining if a certain portion of the cortex is normal. This approach was successful when evaluating MGV with the 14‐ and 21‐days' post injury being totally out of normal range (b). Skewness had a brother difference with 14‐, 21‐, and 30‐days' post TBI being definitively different (c). Kurtosis of the damaged area was still beyond the normal range, but much closer to the normal range compared with MGV and skewness (d)

For this part of the experiment adult rats (30–50 weeks old) were subjected to the same procedure as described of young and aged animals. The difference was that we did not record imaging during the acute phase of TBI in this group and first measurement was done 7 days after the initial injury. Measuring the apparent area of the lesion at 7 days after TBI (47.889 ± 15.093 mm^2^) it was surprisingly similar to measurements obtained 48 hr after the TBI in young (34.675 ± 12.361 mm^2^) and old (40.186 ± 16.775 mm^2^) animal (*p* > .05). However, the apparent damaged area increased over time reaching its peak 21 days after TBI and then decreased toward the end of the experiment, but still larger than the one measured at the beginning of the chronic phase (Figure 4a).

The mean gray value measured at the beginning of the chronic phase revealed a decrease in adults (55.8 ± 1.14) compared with both young (56.26 ± 0.38) and old animals (61.39 ± 0.28) at 48 hr (*p* < .001). This phenomenon continued until day 21, where the lowest value was recorded. At the end of the experiment the MGV increased but remained lower compared with the 7‐day mark (Figure 4b).

In the chronic phase of TBI a much higher asymmetry was recorded of the probability distribution of a real‐valued random variable around its mean, especially when measuring skewness. This asymmetry reached its peak 21 days after TBI and then decreased, at 30 days. In the case of the kurtosis the asymmetry recovered 30 days after the TBI was almost nonexistent.

## 4 DISCUSSION

OCT imagining is based on the concept of interference using low coherence optical radiation (Fujimoto et al., 2000; Popescu et al., 2011). It is able to provide high speeds, microns resolution and, due to the fact that uses optical/infrared radiation, it is a completely noninvasive investigation. This is one of the reasons it has become such an attractive tool to integrate in modern research. However, some technical disadvantages of the method prevent, for the moment, its brother use in clinical setups. We have already reported OCT as a promising tool for brain imaging (Osiac, Balseanu, Mogoanta, et al., 2014), but extensive and detailed investigations for clearer and unambiguous connection with the morphology and physiology of the observed processes, are still to be done (Osiac, Balseanu, Catalin, et al., 2014; Sato et al., 2019). As such we wanted to test its potential in a different neurological disorder, one that can have long‐lasting cellular changes in the brain (Donat, Scott, Gentleman, & Sastre, 2017; McColl et al., 2018; Witcher et al., 2018; Zhao et al., 2018) and a high cost of monitoring (van Dijck et al., 2019), in the hope that, with technological improvements, OCT could become a cheaper and faster alternative to MRI.

TBI usually occurs when a sudden force damages the brain, disrupts its normal function and leads to profound physical, psychological, cognitive, emotional, and social effects (Rowe et al., 2016; Sharma et al., 2020; Zhao et al., 2018). This paper wants to be a proof of concept by investigating if OCT can help with the evaluation and monitoring of severe TBI by using OCT in an experimental setting.

Due to its complexity TBI is classified in primary and secondary injuries. While primary injury is induced by a mechanical force that occurs simultaneously with the initial trauma, the secondary injury may develop in hours to days from the impact and can superimpose with the primary one (Bolton & Saatman, 2014). Also, TBI can be focal or diffuse, although they are commonly found together. While focal injury implies scalp injury, skull fracture and surface contusions, diffuse injury usually imply diffuse axonal injuries, hypoxic–ischemic damage, meningitis, and vascular injury, especially if that TBI is caused by acceleration‐deceleration forces (Bolton & Saatman, 2014). Because secondary and diffuse injuries are extremely difficult to predict, we chose a TBI method that inflicted similar primary and focal damage, thus insuring the secondary one will vary as less as possible. In order to have a more controlled lesion but still keep some of the real‐life characters of TBI, we decided that the internal lamina of the skull should be broken during the trauma itself, instead of making the injury through a total craniotomy. One major disadvantage of OCT, at this time, is that it needs a direct view of the measured tissue and, as such, we needed to expose the brain, thus limiting the power of the study.

To our surprise OCT measurements of the normal contralateral hemispheres revealed that the method is able to detect physiological changes between young and aged animals, at least under the described conditions. This could be just a consequence of the cortex different water content between young and aged animals (Ayrapetyan, Heqimyan, & Nikoghosyan, 2012; Deghoyan, Nikoghosyan, Heqimyan, & Ayrapetyan, 2014), as older animals tended to have a larger MGV detected by OCT imaging.

Because TBI has two distinct stages we evaluated the opportunity of using OCT imaging both in acute and chronic phases. The acute stage, 6 to 48 hr after TBI, was investigated in both young and aged animals. Within this interval the apparent damaged area showed no difference between older animals compared with young ones, except for the 24 hr mark, where older animals had larger apparent damaged areas compared with young animals. Two days after the initial TBI, in the aged animal group, we were able to detect a decrease in the apparent lesion area which could show a delayed response in older animals compared with young ones, as already reported by other groups (Brickler, Morton, Hazy, & Theus, 2018; Kumar et al., 2013; Marker et al., 2010). Due to the fact that our OCT setups cannot discriminate individual cells within the newly form scar, our findings suggest that, at least for the acute phase of the TBI, this technique evaluates water content and not reflected patterns due to cell density, as in this period water metabolism varies faster that cellular migration or proliferation (Wu et al., 2020). When measuring the apparent OCT lesion, no difference was found between young and old animal 48 hr after the initial TBI. Furthermore, adults displayed a similar apparent lesion area detected by OCT 7 days after TBI suggesting that there was a relatively stable interval, at least for adult animals, after the acute phase of the lesion. Data collected by OCT imaging suggested a steady increase of altered area noticed 2 weeks after the lesion. This phenomenon had a peak at 3 weeks after the TBI, detected by all analyzed OCT parameters, then dropping 30 days after lesion induction. This drop in mean gray value could be due to an increase in newly formed blood vessels, after the massive loss caused by the TBI itself (Salehi, Zhang, & Obenaus, 2017) and/or could indicate an existing secondary lesion, as other experimental setups have reported an increase of the damaged area in the first 30 days, but using a different experimental design (Xuan et al., 2013). For the most part of the acute period, the mean gray value of the scanned area showed an increase in old animals compared with younger ones after TBI. The fluctuating levels in light scattering in the young animals could also be linked to a more dynamic response to damage in this group compared with the aged one (Onyszchuk et al., 2008). The value of pixel intensity is depending on the scatter properties of the infrared light, but it had not been linked to a cellular response in any experiments that we know of. While the damaged area increased for both groups at 12 hr after TBI, the intensity of the mean value decreased—probably due to a rapid response in repairing processes (additional angiogenesis and/or cellular regeneration) some of the initial damage being repaired/contained; at 24 hr MGV values are similar in the two groups, presumably for different reasons: young animals probably had an earlier and more robust cell migration towards the lesion sight, while in old subjects reparatory processes probably started later, at 24 hr and not around 12 hr as seen in young individuals.

In the chronic part of this experiment the mean gray value has decreased for the first 3 weeks and returned to similar values to the contralateral brain tissue 30 days after the TBI. Interestingly MGV had a negative pick around 3 weeks after the trauma coinciding to a period reported to have some of the lowest blood vessel diameters following TBI (Salehi et al., 2017; Siddiq et al., 2012). Looking at similar reports it is tempting to say that MGV is directly correlated to blood vessel diameters. However, if this could be true for the chronic phase, for the acute one probably different processes (membrane shattering, tissue edema, and cell density) contribute to overall changes that appear in pixel intensity values.

## 5 CONCLUSIONS

As technology improves, less invasive and non‐harmful technologies will be used either to evaluate patients and/or to establish their outcome. As light penetration will improve, OCT could start being used to evaluate deeper tissues in a rapid and safe manner. Although in the present study we still used an extremely invasive approach, we were able to show that OCT could be used as an imaging tool in determining acute changes of the cortex after TBI. Furthermore, we could detect fluctuation from normal during the chronic phase. As all the research done was on pixels and not on real tissue, the analyzed parameters (mean, skewness, and kurtosis) of the detected pixel intensity could represent the bases of an objective way to detect changes within the brain structure after injuries. With further advances, a clear correlation between OCT parameters and biological processes could be achieved, and OCT could prove to be an important and non‐expensive tool in evaluating patients suffering from TBIs.

## Supporting information


**Figure S1**
